# Effects of bovine tumor necrosis factor alpha decoy receptors on cell death and inflammatory cytokine kinetics: potential for bovine inflammation therapy

**DOI:** 10.1186/s12917-019-1813-0

**Published:** 2019-02-28

**Authors:** Sotaro Fujisawa, Satoru Konnai, Tomohiro Okagawa, Naoya Maekawa, Akina Tanaka, Yasuhiko Suzuki, Shiro Murata, Kazuhiko Ohashi

**Affiliations:** 1 Department of Disease Control, Faculty of Veterinary Medicine, Hokkaido University, Sapporo, Hokkaido 060-0818 Japan; 2 Department of Advanced Pharmaceutics, Faculty of Veterinary Medicine, Hokkaido University, Sapporo, 060-0818 Japan; 3 Division of Bioresources, Research Center for Zoonosis Control, Hokkaido University, Sapporo, 001-0020 Japan; 4 Global Station for Zoonosis Control, Global Institution for Collaborative Research and Education (GI-CoRE), Hokkaido University, Sapporo, 001-0020 Japan

**Keywords:** Cattle, TNF-α, TNF receptor, Decoy receptor, Cell death, Inflammatory cytokines

## Abstract

**Background:**

Refractory diseases, including bacterial infections, are causing huge economic losses in dairy farming. Despite efforts to prevent and treat those diseases in cattle, including the use of antimicrobials, it is not well controlled in the field. Several inflammatory cytokines, including tumor necrosis factor alpha (TNF-α), play important roles in disease progression; thus, blocking these cytokines can attenuate the acute and sever inflammation and may be a novel strategy for treatment. However, biological drugs targeting inflammatory cytokines have not been used in cattle. Therefore, in this study, bovine sTNFR1 and sTNFR2 IgG1 Fc-fusion proteins (TNFR1-Ig and TNFR2-Ig) were produced, and their anti-inflammatory functions were analyzed in vitro, to develop decoy receptors for bovine TNF-α.

**Results:**

Both TNFR1-Ig and TNFR2-Ig were shown to bind with TNF-α, and TNFR2-Ig showed higher affinity toward TNF-α than TNFR1-Ig. We next stimulated murine fibroblast-derived cells (L929 cells) with TNF-α to induce cell death and analyzed cell viability in the presence of TNFR-Ig proteins. Both TNFR1-Ig and TNFR2-Ig suppressed TNF-α-induced cell death, significantly improving cell viability. In addition, cell death induced by TNF-α was suppressed, even at low TNFR2-Ig concentrations, suggesting TNFR2-Ig has higher activity to suppress TNF-α functions than TNFR1-Ig. Finally, to examine TNFR2-Ig’s anti-inflammatory, we cultured peripheral blood mononuclear cells from cattle with TNF-α in the presence of TNFR2-Ig and analyzed the gene expression and protein production of the inflammatory cytokines IL-1β and TNF-α. TNFR2-Ig significantly reduced the gene expression and protein production of these cytokines. Our results suggest that TNFR2-Ig inhibits inflammatory cytokine kinetics by blocking TNF-α to transmembrane TNFR, thereby attenuating excessive inflammation induced by TNF-α.

**Conclusions:**

Collectively, the findings of this study demonstrated the potential of TNFR2-Ig as a novel therapeutic for inflammatory diseases, such as bovine clinical mastitis. Further investigation is required for future clinical application.

## Background

In dairy farming and the livestock industry worldwide, numerous infectious diseases threaten animal health and productivity. There are effective controlling strategies, such as vaccination, for some diseases. However, various refractory diseases, including bacterial infections such as paratuberculosis, mycoplasmosis, and mastitis remain serious issues [1, 2]. In these diseases, inflammation is a common feature, and many types of inflammatory cytokines, such as interleukin (IL)-1, IL-6, and tumor necrosis factor alpha (TNF-α) have been strongly associated with disease progression [3, 4]. Although the current treatment for these inflammatory bacterial diseases mainly depends on antibiotics, many issues remain to be resolved. While antibiotics are typically effective for certain bacteria, if applied adequately, sometimes they are not effective because the bacteria have developed a mechanism to escape from the lethal effect of antimicrobials. Moreover, the improper use of antibiotics causes environmental problems, such as antibiotic-resistant bacteria and milk pollution [5]. In European countries, predonisolone, one of the steroids, are used to reduce inflammation [6]. However, it has been indicated that steroid administration reduce milk production and decrease prolactin release [7]. Therefore, developing novel therapeutic methods for these refractory bacterial infections is required.

As mentioned, inflammatory cytokines are critical factors in bacterial infection progression. Thus, inhibiting these cytokines may be promising as part of an overall control strategy. TNF-α is produced by fibroblasts and epithelial cells as well as activated immune cells. Immediately upon microbial invasion, it serves various functions, such as activation of immune cells and cytotoxicity to tumor cells [8, 9]. TNF-α constitutes a trimer and is expressed on cell membranes [9, 10]. Membrane-expressed TNF-α (mTNF-α) is cleaved by a TNF-α converting enzyme and released into the bloodstream as soluble TNF-α (sTNF-α) [11]. The receptors of TNF-α are classified as type 1 (TNFR1) and type 2 (TNFR2). While TNFR1 is expressed on various cell types, the existence of TNFR2 is limited to immune and epithelial cells [8, 9]. Extracellular domains of both receptors contain similar motifs, including cysteine-rich regions where TNF-α binds. While TNFR1 can be activated by both mTNF-α and sTNF-α, activation of TNFR2 is mediated by only mTNF-α, despite the fact that sTNF-α can also bind TNFR2 [9, 12]. The intracellular domain of TNFR1 contains a death domain (DD); in response to TNF-α binding, the TNFR1-associated death domain protein (TRADD) interacts with DD immediately. There are two different cascades known to be downstream of TRADD. In the canonical pathway, TRADD interacts with various adaptor molecules, such as Receptor-Interacting serine/threonine-Protein Kinase 1 (RIPK1) and TNF receptor associated factor 2 (TRAF2), resulting in NF-κB activation [13]. NF-κB promotes the transcription not only of cytokine genes, including IL-1 and TNF-α, but also of Cellular Inhibitor of Apoptosis Protein 1 (c-IAP1) and c-IAP2 to prevent apoptosis-inducible pathways [14–16]. In contrast, in the non-canonical pathway, the Fas-associated DD protein (FADD) interacts with TRADD and induces apoptosis via Caspase 8 and 10 activation [13]. On the other hand, regarding TNFR2, TRAF2 interacts directly with TNFR2’s intracellular domain upon mTNF-α-stimuli. This process then leads to NF-κB activation, regulatory T cells proliferation, and cytotoxic T cell function suppression [17, 18]. Accordingly, TNF-α greatly contributes to the pathogenesis of inflammatory diseases.

On the contrary, soluble TNFRs (sTNFRs) might reduce inflammation as “decoy receptors” by competitively inhibiting TNF-α/membrane TNFR (mTNFR) interactions [19]. sTNFRs consist of only the extracellular domains of TNFRs, which are cleaved from the membrane by proteases and released into the bloodstream. In inflammatory diseases, the concentrations of sTNFR in the bloodstream appear to increase in correlation with disease progression [20, 21]. Therefore, sTNFRs should suppress excess immune responses and inflammation.

In human inflammatory diseases, such as rheumatoid arthritis, inhibiting TNF-α with anti-TNF-α antibodies or sTNFR-fusion proteins has been gaining increasing attention as a promising treatment strategy. For example, sTNFR2-fusion proteins are widely used for rheumatoid arthritis, with high therapeutic performance [22–24]. However, in the veterinary field, few reports exist of therapeutic strategies targeting inflammatory cytokines and the application of biologicals. In this study, Fc-fusion proteins of bovine sTNFR were developed, and their anti-apoptotic and anti-inflammatory properties were evaluated in vitro using recombinant bovine TNF-α based assays.

## Results

### Generation of recombinant bovine TNFR-Ig and Cont-Ig

**Fig. 1:**
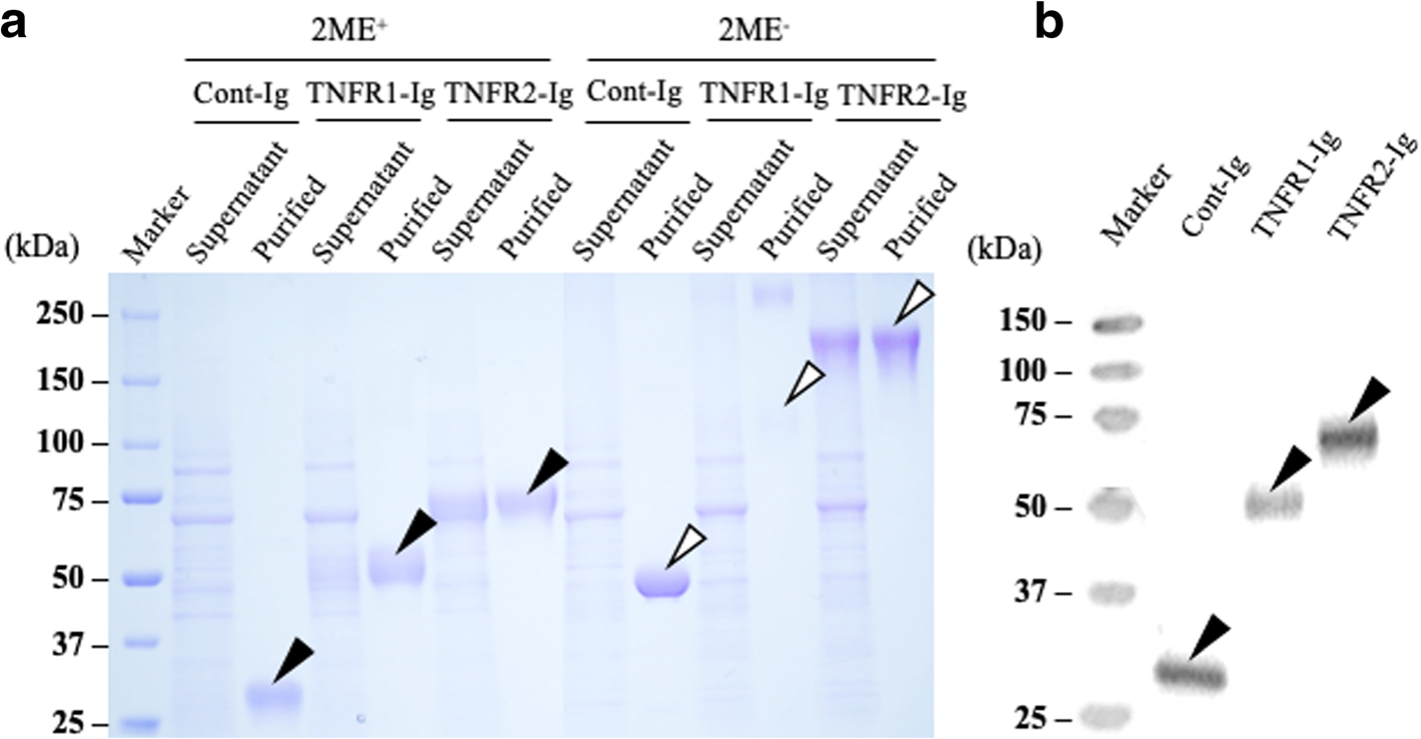
Generation of recombinant bovine TNFR1-Ig and TNFR2-Ig (**a**) SDS-PAGE, (**b**) Western blot analysis of purified proteins using anti-bovine IgG antibody

To evaluate the TNF-α decoy receptor’s efficacy for bovine inflammatory diseases, we first prepared recombinant bovine TNFR-Ig, consist of the extracellular domains of bovine TNFR1 or 2 and the Fc domain of bovine IgG1. In addition, as a negative control, Cont-Ig, consists of the signal peptide domain of bovine TNFR2, and the Fc domain of bovine IgG1, was generated using the Expi293 Expression System. Figure 1 shows the expressions and purities of each recombinant protein, as determined by SDS-PAGE and Western blotting with the anti-bovine IgG Fc antibody (Fig. 1a and b). TNFR1-Ig, TNFR2-Ig, and Cont-Ig were detected at approximately 50, 70, and 30 kDa, respectively. Each recombinant protein was detected at double the molecular weight in the absence of 2ME, confirming that these proteins formed dimers (Fig. 1b).

### Binding of TNFR-Igs to bovine TNF-α

**Fig. 2:**
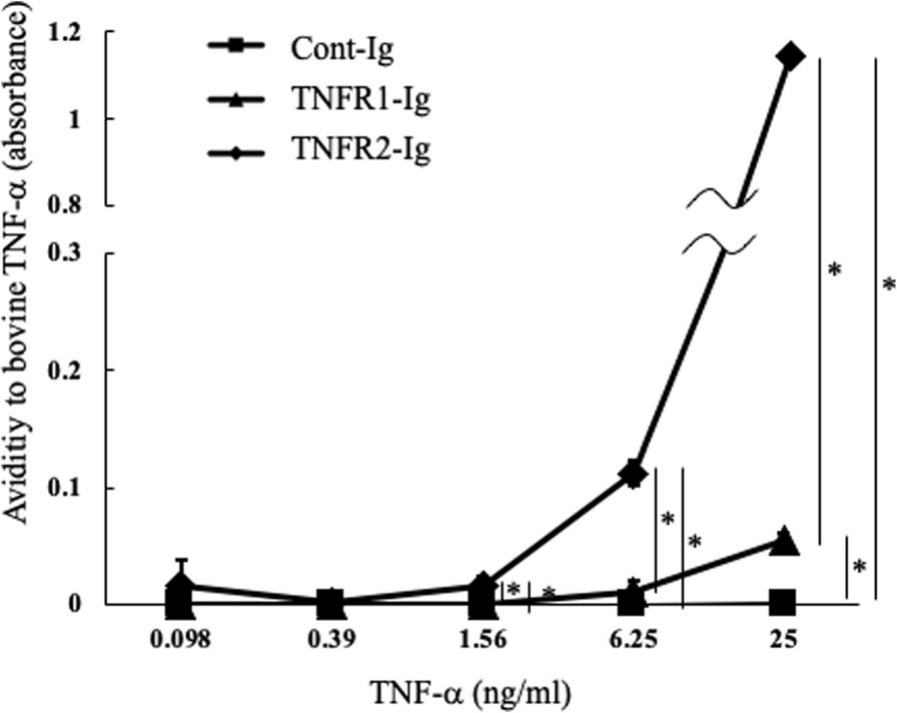
Avidities of TNFR1 and R2-Ig to bovine TNF-α The avidities of TNFR-Ig to bovine TNF-α were analyzed using ELISA using biotinylated anti-bovine TNF-α antibody. Briefly, TNFR-Igs or Cont-Ig was fixed on ELISA plate and recombinant bovine TNF-α (0.098–25 ng/ml) was added. After the incubation (room temperature) and washing, biotinylated anti-bovine TNF-α antibody was added. TNFR2-Ig showed significantly higher avidity to bovine TNF-α than that showed by TNFR1-Ig (*n* = 3), * *p* < 0.05

The binding of TNFR1-Ig and TNFR2-Ig to bovine TNF-α was analyzed by enzyme-linked immunosorbent assay (ELISA). TNF-α-dose dependent increases in absorbance were observed with TNFR1-Ig and TNFR2-Ig application, whereas changes in absorbance were not detected when using Cont-Ig, indicating that both TNFR1-Ig and TNFR2-Ig can capture bovine TNF-α (Fig. 2). In addition, TNFR2-Ig showed 11-fold at 6.25 ng/mL of TNF-α and 21-fold absorbance at 25 ng/mL of TNF-α compared to TNFR1-Ig, suggesting that TNF-α’s affinity toward TNFR2-Ig was higher than that of TNFR1-Ig.

### Effect of TNFR-Igs on cell death by TNF-α

**Fig. 3:**
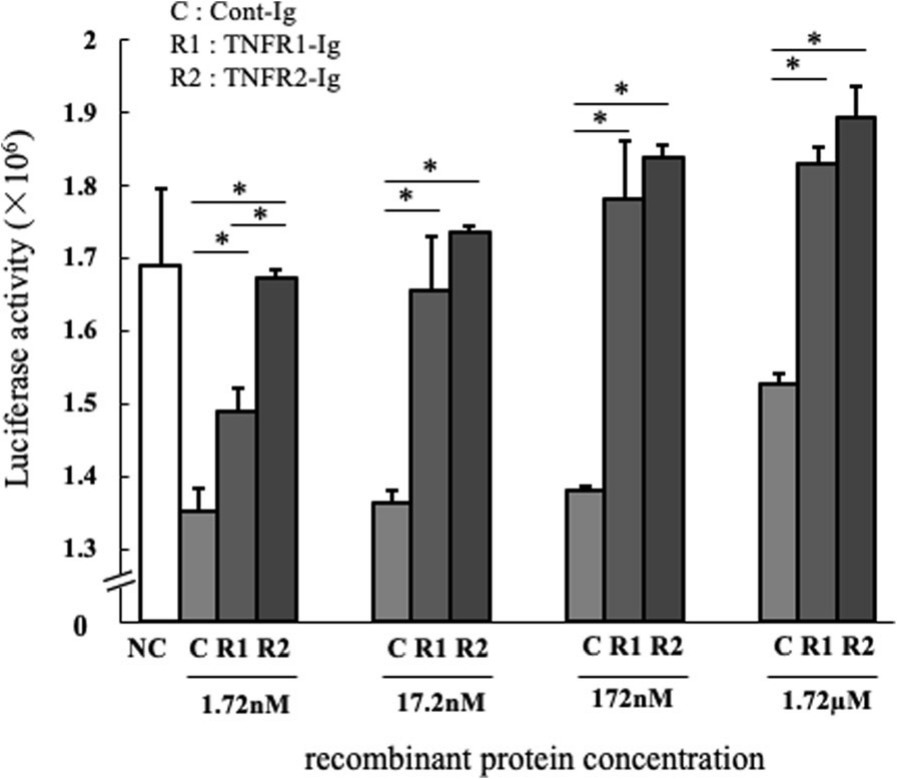
Effect of TNFR-Igs on cell death by TNF-α L929 cells were cultured with recombinant bovine TNF-α, actinomycin-D, and TNFR1-Ig, TNFR2-Ig, or Cont-Ig under several concentrations. Cell viabilities were assessed using the RealTime-Glo™ MT Cell Viability Assay according to the manufacturer’s instructions. Both TNFR-Ig, but particularly TNFR2-Ig, significantly reduced cell death induced by TNF-α. The assays were conducted in triplicate. * *p* < 0.05

TNF-α induces cell death by activating Caspase 8 and 10, via the DD, when captured by mTNFR1, which is expressed on cell membranes [9]. Therefore, we investigated whether TNFR-Ig could suppress cell death in L929 cells and bovine PBMCs driven by TNF-α using the Realtime-Glo MT Cell Viability Assay. In L929 cells, treatment with TNFR1-Ig and TNFR2-Ig significantly enhanced cell viability compared to Cont-Ig; cell viability of TNFR2-Ig-treated L929 cells tended to be higher than those of TNFR1-Ig-treated cells, indicating that TNFR2-Ig could suppress cell death by capturing TNF-α more efficiently than TNFR1-Ig (Fig. 3). In bovine PBMCs, however, stimulation with TNF-α did not induce cell death at all, and TNFR-Ig treatment did not affect cell viability (data not shown). Considering these results, TNFR2-Ig appears to have higher affinity toward TNF-α and can inhibit TNF-α function compared to TNFR1-Ig in L929 cells. Therefore, we proceeded with experiments only using TNFR2-Ig.

### Effect of TNFR2-Ig on inflammatory cytokine kinetics by TNF-α

**Fig. 4:**
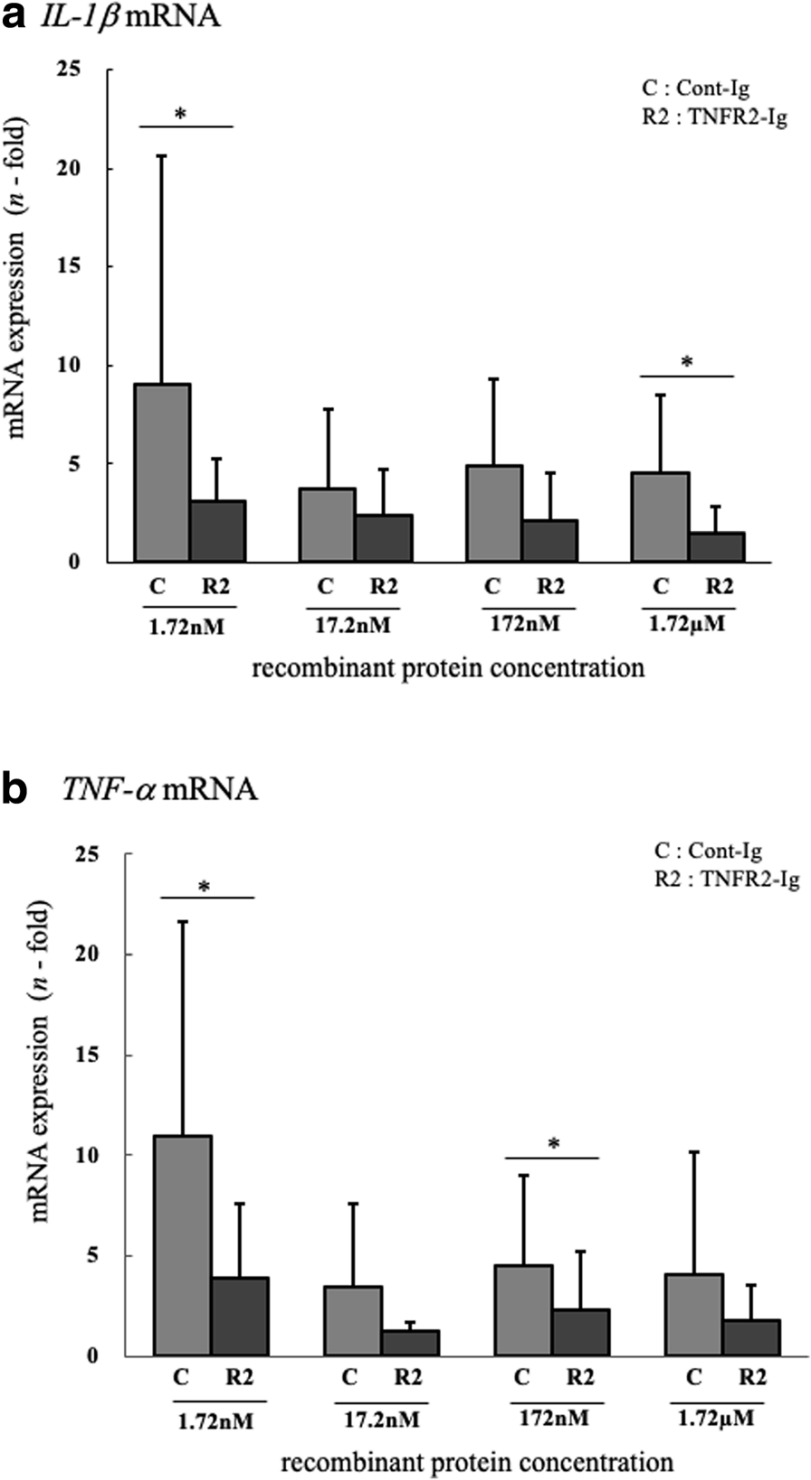
Effect of TNFR2-Ig on TNF-α-induced inflammatory cytokine gene expressions PBMCs were cultured with recombinant bovine TNF-α, actinomycin-D, and TNFR2-Ig or Cont-Ig. The gene expression of (**a**) IL-1β mRNA and (**b**) TNF-α mRNA in bovine PBMCs were assessed using real-time PCR. The results of IL-1β and TNF-α mRNA expression are presented as a ratio to their expressions in untreated PBMCs (*n* = 6). * *p* < 0.05

**Fig. 5:**
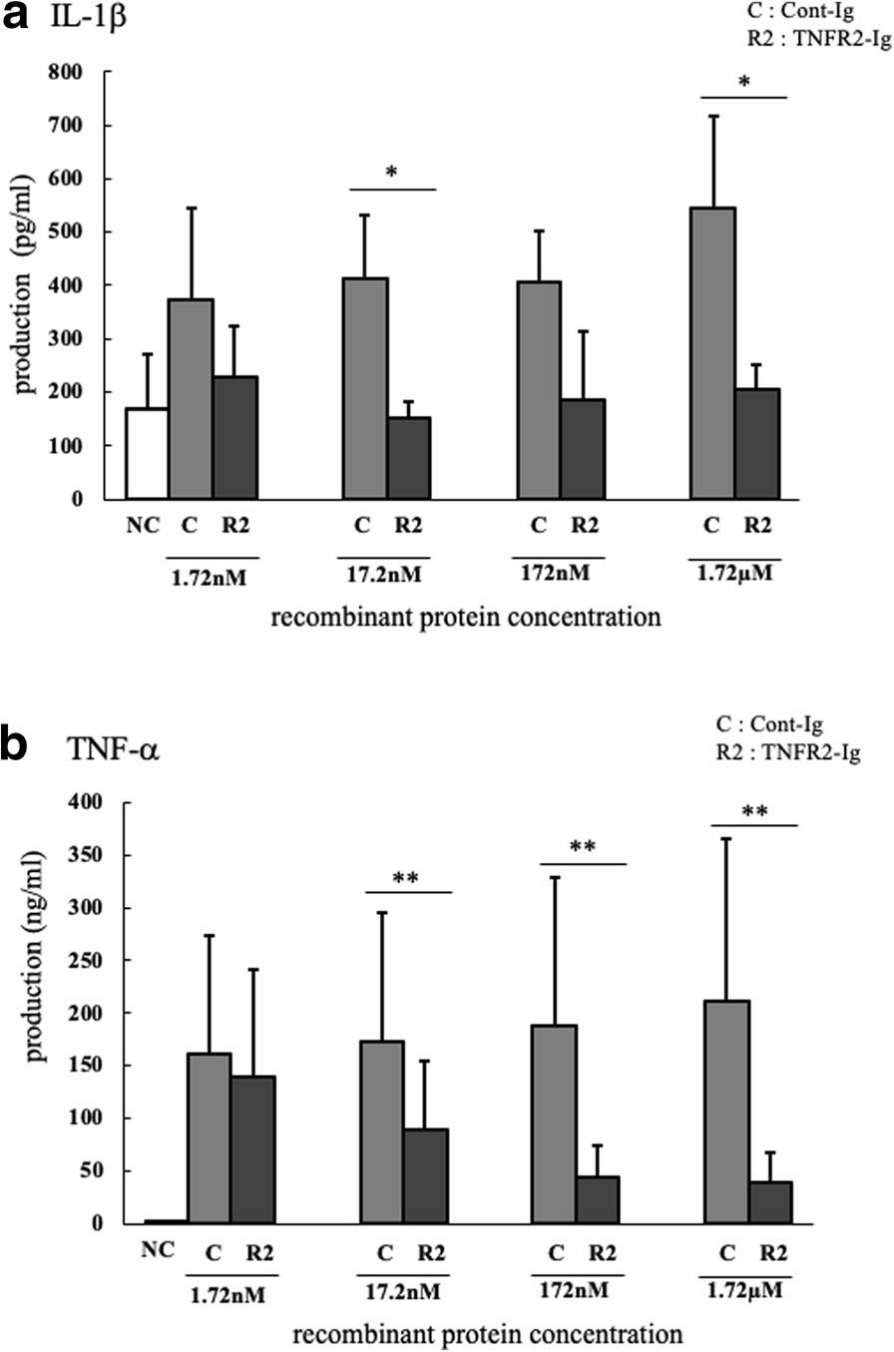
Effect of TNFR2-Ig on TNF-α-induced inflammatory cytokine productions. PBMCs (*n* = 6) were cultured with recombinant bovine TNF-α, actinomycin-D, and TNFR2-Ig, or Cont-Ig under several concentrations. IL-1β and TNF-α in the cultural supernatant were assessed using ELISA. TNFR2-Ig significantly suppressed the production of both cytokines induced by bovine TNF-α. ** *p* < 0.01, * *p* < 0.05

It has been reported that TNF-α can induce not only cell death but also NF-κB activation when captured by mTNFR1 and mTNFR2 [14], which can lead to inflammatory cytokine production. Therefore, we investigated whether TNFR2-Ig could reduce the gene expression and production of inflammatory cytokines from bovine PBMCs, driven by TNF-α. Treatment with TNFR2-Ig significantly reduced both gene expression and protein production of IL-1β and TNF-α, induced by stimulation with TNF-α (Figs. 4 and 5). These results indicated that TNFR2-Ig trapped soluble TNF-α and competitively inhibited the interaction between TNF-α/mTNFR, and could modulate the inflammatory response triggered by TNF-α.

## Discussion

TNF-α is produced immediately upon pathogen-invasion, including bacterial infections, and induces various immune responses such as cell proliferation and inflammation [8, 9]. It has been previously reported that, in the veterinary field and particularly in bovine infectious diseases, numerous bacteria such as *Escherichia coli* and *Mycoplasma bovis* can induce the prompt release of TNF-α [25]. In human clinical medicine, soluble TNFR (sTNFR) seems capable of suppressing TNF-α bioactivities by competitively inhibiting TNF-α/membrane TNFR (mTNFR) interactions. In this study, we established soluble bovine TNFRs Fc-fusion proteins (TNFR-Ig) and demonstrated that these proteins possess these inhibitive features as well as the potential to be novel therapeutic treatments for the inflammatory diseases mentioned above.

In our experiments, we showed that both TNFR1-Ig and TNFR2-Ig can capture bovine TNF-α, and that TNFR2-Ig has much higher affinity toward TNF-α than TNFR1-Ig. According to previous reports, the affinities of human TNF-α and TNFR are still controversial. In some reports, TNFR1 seemed have greater affinity toward TNF-α than TNFR2 [26], while there have also been opposite suggestions [27]. These contradictions may depend on whether TNF-α and TNFR are membrane-expressed or in their soluble form. Regarding human mTNFR, it has been reported that mTNFR1 was higher in affinity toward sTNF-α than mTNFR2 [28]. However, there is little information of the affinities between sTNFR and sTNF-α. In this study, regarding bovine sTNFR, the affinity toward sTNF-α seemed much higher for sTNFR2 than for sTNFR1. Nevertheless, we only measured the bindings of sTNFRs and sTNF-α by ELISA, so further analyses, such as evaluation of bonding and dissociation constants, are required. Moreover, additional experiments using mTNF-α are needed to evaluate whether TNFR-Ig can inhibit mTNF-α as well as sTNF-α.

When TNF-α binds mTNFR1, Caspase 8 and 10 are activated via the DD, resulting in apoptosis [13]. While both TNFR1-Ig and TNFR2-Ig, and particularly TNFR2-Ig, significantly reduced cell death in L929 cells triggered by TNF-α, regarding bovine PBMCs, neither TNF-α or TNFR-Ig affected cell viabilities at all. To explain these different responses between L929 cells and PBMCs, we present two hypotheses. The first is that this is because of the difference of mTNFR1 functions on each cell. L929 cells have been reported to be very susceptible to the cytotoxicity of TNF-α, and generally used for functional analysis of TNF-α [29, 30]. When TNF-α binds to mTNFR1, it promotes the formation of the death domain/TRADD complex. Typically, this complex would activate NF-κB via recruitment of other adaptor molecules such as RIPK1 and TRAF2, which induces inflammatory responses or cell proliferations [13]. However, in some cases, although the mechanisms are still unclear, the death domain/TRADD complex induces apoptosis via activation of caspases caused by RIP1K ubiquitination deficiency [31, 32]. Although TNFR1’s cell type-dependent functions are poorly understood, we might uncover the mechanisms underlying the different responses between L929 cells and PBMCs by analyzing the activation of downstream pathways of the death domain/TRADD complex. The second hypothesis focuses on the receptor types expressed on each cell. While only mTNFR1 is expressed on L929 cells, PBMCs express both mTNFR1 and mTNFR2 [8, 9]. When mTNF-α captured TNF-α, it activates NF-κB and promotes the transcriptions of c-IAP1 and c-IAP2, which result in the suppression of apoptosis-inducible pathway [15, 16], hence cell death triggered by mTNFR1 was mediated. Further experiments, such as expression analysis of mTNFR, or the evaluation of the activities of related molecules like Caspase 8, NF-κB, or c-IAP, are warranted to verify these hypotheses.

As previously described, TNF-α can trigger cytokine gene transcriptions, including IL-1, IL-6, and TNF-α via activation of NF-κB. We demonstrated that both gene expressions and productions of IL-1β and TNF-α in PBMCs triggered by the stimulation of TNF-α were significantly reduced by treatment of TNFR2-Ig, indicating that TNFR2-Ig can mediate inflammatory responses driven by TNF-α by competitively inhibit TNF-α/mTNFR interactions.

In this study, we proposed the potential of TNFR2-Ig to be a novel method to control inflammatory diseases in cattle. However, our experiments were based on recombinant bovine TNF-α; thus, our results could not be necessarily applied to infection situations because disease progressions are mediated by more complex interactions between pathogens and host immunity. Hence, further experiments using bacterial antigens or live bacteria, and bovine mammary epithelial cells are required to support TNFR2-Ig’s efficacy. Moreover, TNFR2-Ig’s function is no more than suppressing inflammation and diseases are not eradicable by TNFR2-Ig treatment alone because the target of this approach is not pathogen elimination. Hence, the administration of TNFR2-Ig would be for attenuation of acute and severe inflammation, such as *E. coli*-mediated peracute mastitis. On the other hand, the immunosuppressive effect of TNFR2-Ig is limited to the inhibition of TNF-α, and should be much more moderate than that driven by other immunosuppressive agents, such as steroids. Considering clinical application, therefore, careful and further experiments are required to ascertain the therapeutic potential. In addition, combining TNFR2-Ig with existing approaches such as antibiotics should be investigated.

## Conclusions

Collectively, this study indicates that both TNFR1-Ig and TNFR2-Ig were shown to bind with TNF-α, and TNFR2-Ig showed higher affinity toward TNF-α than TNFR1-Ig. Both TNFR1-Ig and TNFR2-Ig suppressed TNF-α-induced cell death and significantly improved cell viabilities. Interestingly, cell death induced by TNF-α was suppressed even in low concentrations of TNFR2-Ig, suggesting that TNFR2-Ig has higher activity to suppress the TNF-α functions than TNFR1-Ig. Furthermore, TNFR2-Ig significantly reduced inflammatory cytokine genes’ expressions and their protein production. These results suggest that TNFR2-Ig inhibits inflammatory cytokine kinetics via blocking of TNF-α to transmembrane TNFR, and thereby, attenuate excessive inflammation induced by TNF-α. Overexpression of TNF-α is a major death-causing factor in several bovine inflammatory diseases. This study demonstrated TNFR2-Ig’s potential as a novel therapeutic for the bovine inflammatory diseases such as mastitis, arthritis, and pneumonia. Further investigations are required for clinical application in the future.

## Methods

### Cell cultures

Expi293F™ cells^1^ (A14635) were cultured in Expi293™ Expression Medium1 at 37 °C, 125 rpm, and 8% CO_2_. Murine fibroblast-derived L929 cells^1^ (ATCC CCL-1) were cultured in low glucose Dulbecco’s Modified Eagle Medium (D-MEM)^1^ containing 10% heat-inactivated fetal bovine serum (FBS)^2^ at 37 °C and 5% CO_2_. Bovine peripheral blood mononuclear cells (PBMCs) were isolated from the venous blood of healthy cattle (maintained at the Field Science Center for Northern Biosphere, Hokkaido University) by density gradient centrifugation in Percoll solution.^3^ Purified PBMCs were cultured in RPMI medium^4^ containing 10% heat-inactivated FBS, 0.01% L-glutamine, 200 U/mL penicillin, and 200 μg/mL streptomycin^5^ at 37 °C and 5% CO2.

### Cloning of cDNA encoding the extracellular domain fragment of bovine TNFR1 and TNFR2

**Fig. 6:**
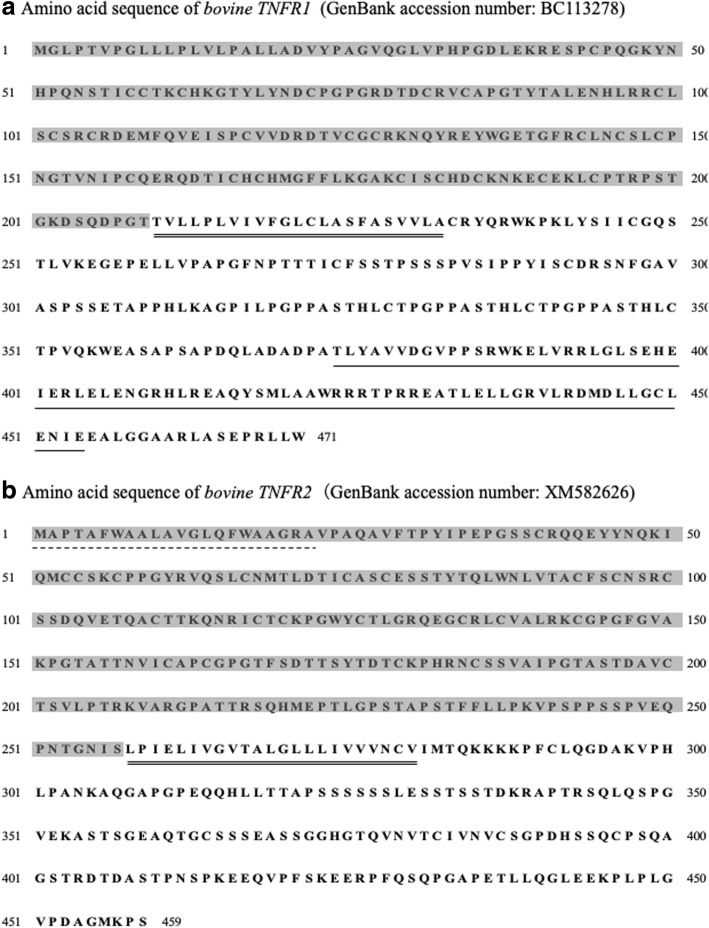
Amino acid sequence of bovine TNFR1 and TNFR2 genes. Amino acid sequence of (**a**) Bovine TNFR1 and (**b**) bovine TNFR2 genes are shown. The single underline in TNFR1 indicates the DD. The dotted line in TNFR2 indicates a signal peptide. Transmembrane regions are indicated using double line. Predicted extracellular domains are shadowed

Total RNA was extracted from the isolated PBMCs of healthy cattle using TRI reagent®^6^ according to the manufacturer’s instructions. Remnant DNA was removed from the RNA samples with DNase I (amplification grade)^5^ treatment for 10 min at 65 °C. The cDNA was synthesized using 1 μg of RNA with PrimeScript Transcriptase^7^ according to the manufacturer’s instructions. The signal peptide and the extracellular domains of the bovine TNFR1 and TNFR2 genes were predicted using the software systems SignalP (http://www.cbs.dtu.dk/services/SignalP/) and SOSUI (http://harrier.nagahama-i-bio.ac.jp/sosui/sosui_submit.html) respectively, based on the sequences registered in the GeneBank database (BC113278 and NM001040490) (Fig. 6). The extracellular domain fragments of bovine TNFR1 and TNFR2 and the signal peptide domain of TNFR2 cDNA (as a negative control) were amplified by PCR using the specific primers shown in Table 1. The cycling conditions consisted of initial denaturation at 94 °C for 5 min, followed by 40 cycles of 94 °C for 15 s, 55–45 °C at the rate of 1.0 °C/cycle for 30 s, and 68 °C for 1 min, and final extension at 68 °C for 7 min. Each PCR amplicon was inserted into the cloning site of a modified pCXN2.1 (+) - bovine IgG1 Fc plasmid (kindly provided by Dr. Takehiko Yokomizo, Juntendo University, Tokyo, Japan) that contained the Fc fragment of bovine IgG1 at the C terminal, and transformed into a competent HST08 *E.coli* strain. Recombinant plasmids were purified with the FastGene Xpress Plasmid PLUS Kit,^8^ according to the manufacturer’s instructions. The recombinant plasmids were named pCXN2.1 (+) - TNFR1-Ig, pCXN2.1 (+) - TNFR2-Ig, and pCXN2.1 (+) - Cont-Ig.

**Table 1:**
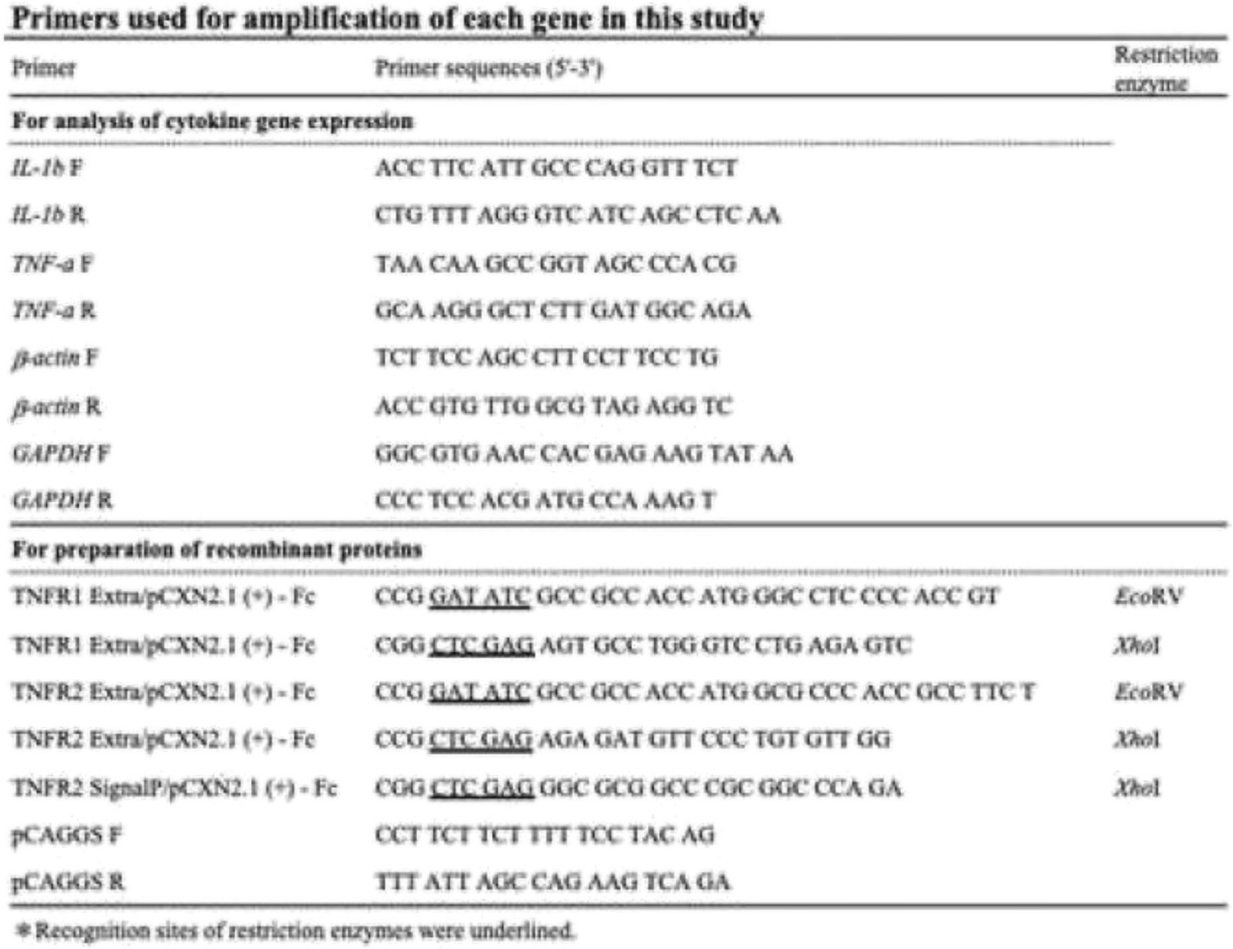
Primers used for amplification of each gene in this study

### Expression of recombinant soluble bovine TNFR1-Ig and TNFR2-Ig fusion proteins

Recombinant plasmids were transfected into maintained Expi293F™ cells using ExpiFectamine™ 293 Reagent^1^, ExpiFectamine™ 293 Transfection Enhancer I^1^, and II^1^ according to the manufacturer’s instructions. Culture supernatants containing TNFR-Ig or Cont-Ig were harvested for 7 days after transfection, and the recombinant proteins were purified with Ab-Capture ExTra™^.^^9^ The purified proteins were then concentrated by ultrafiltration using Amicon Ultra.^10^ To confirm TNFR-Ig and Cont-Ig expression, Western blot analysis was performed using the peroxidase-labeled anti-bovine IgG Fc rabbit antibody,^11^ and purities were analyzed by SDS-PAGE. The quantities of the recombinant proteins were determined by sandwich ELISA with the anti-bovine IgG Fc rabbit antibody^11^ and the peroxidase-labeled anti-bovine IgG Fc rabbit antibody^11^.

### Bovine TNF-α binding assay

The binding of TNFR-Ig to bovine TNF-α was analyzed using the anti-bovine TNF-α antibody. Briefly, TNFR-Ig or Cont-Ig was fixed on 96-well plates for ELISA^12^ for 24 h with phosphate buffered saline (PBS). After washing each well three times with PBS, incubation buffer [IB; PBS containing 0.05% Tween 20 and 1% Bovine Serum Albumin (BSA)^4^] was added and incubated for 1 h. The solution was then washed five times with PBS containing 0.05% Tween 20 (PBS-T), and the recombinant bovine TNF-α was added (0.098 ng/mL-25 ng/mL).^13^ After 2 h incubation, samples were washed five times with PBS-T and reacted with the biotinylated anti-bovine TNF-α rabbit antibody^13^ for 1 h. After washing for five times with PBS-T, peroxidase-labeled Pierce™ NeutrAvidin™ Protein^1^ was added and incubated for 1 h. Finally, the mixture was washed five times with PBS-T and reacted with the TMB one component substrate^14^ for 10 min in the dark. The reaction was quenched with 0.18 M H_2_SO_4_ and the absorbance was measured at 450 nm. Each incubation was performed at room temperature. The assay was performed in triplicate.

### Cell viability assay

L929 cells (3 × 10^4^/0.2 mL) were pre-cultured for 24 h on LIMITRAC® 96-well plates.^15^ After cultivation, cells were cultured 24 h with recombinant bovine TNF-α [300 μg/mL (17.2 nM)], actinomycin-D (1 μg/mL)^4^ and TNFR1-Ig or TNFR2-Ig or Cont-Ig at varying concentrations (1.72 nM, 17.2 nM, 172 nM, 1.72 μM). Cell viabilities were measured using the RealTime-Glo™ MT Cell Viability Assay^16^ according to the manufacturer’s instructions. The assay was performed in triplicate.

### Expression analysis of bovine interleukin-1β and TNF-α mRNA

PBMCs (1.5 × 10^6^/0.2 mL) from 6 healthy cattle were cultured for 4 h with recombinant bovine TNF-α [300 μg/mL (17.2 nM)], actinomycin-D (1 μg/mL)^4^ and TNFR1-Ig or TNFR2-Ig or Cont-Ig at varying concentrations (1.72 nM, 17.2 nM, 172 nM, 1.72 μM). As a calibrator sample, PBMCs from each cattle were cultured in the absence of TNF-α, actinomycin-D, and TNFR2-Ig or Cont-Ig. Total RNA from cultured PBMCs was extracted and used to synthesize cDNA as described above. Real-time quantitative RT-PCR was performed using the LightCycler480® SystemII^17^ and SYBR® Premix DimerEraser™ ^7^ according to the manufacturer’s instructions. The primers and target genes are shown in Table 1. The cycling conditions consisted of initial denaturation at 95 °C for 30 s, followed by 45 cycles of 95 °C for 5 s, 60 °C for 30 s and 72 °C for 30 s. A final melting curve analysis was performed from 65 °C to 95 °C at a rate of 0.1 °C/sec (continuous acquisition) to evaluate each primer pair for specificity to confirm that only one product was amplified. Each sample was tested in duplicate, and the relative expression level of IL-1β and TNF-α mRNA were analyzed using the 2^-ΔΔCT^ method [33].

### IL-1β and TNF-α ELISA

PBMCs (5 × 10^5^/0.2 mL) from 6 healthy cattle were cultured for 24 h with recombinant bovine TNF-α [300 μg/mL (17.2 nM)], actinomycin-D (1 μg/mL)^4^ and TNFR1-Ig or TNFR2-Ig or Cont-Ig at varying concentrations (1.72 nM, 17.2 nM, 172 nM, 1.72 μM). IL-1β and TNF-α in the cultural supernatant were measured by ELISA using IL-1beta ELISA Reagent Kit, bovine^1^ and TNF-α Bovine TNF alpha Do-It-Yourself ELISA^13^, respectively, according to the manufacturer’s instructions.

### Statistics

The differences between the groups were examined using the Wilcoxon signed rank test. For multiple group comparisons, the Steel-Dwass’ test or Tukey’s test was performed depending on the sample numbers. A *p*-value less than 0.05 was considered statistically significant.

## Abbreviations

BSA: Bovine Serum Albumin
c-IAP1: Cellular Inhibitor of Apoptosis Protein 1
DD: Death domain
D-MEM: Dulbecco’s Modified Eagle Medium
ELISA: Enzyme-linked immunosorbent assay
FADD: Fas-associated death domain protein
FBS: Fetal bovine serum
IL: Interleukin
mTNFR: Transmembrane TNFR
mTNF-α: Membrane-expressed TNF-α
PBMC: Peripheral blood mononuclear cells
PBS: Phosphate buffered saline
PBS-T: PBS containing 0.05% Tween 20
RIPK1: Receptor-Interacting serine/threonine-Protein Kinase 1
sTNFR2: Soluble TNF receptor 2
sTNF-α: Soluble TNF-α
TNFR1: Associated death domain protein
TNFR1: TNF receptor 1
TNFR2: TNF receptor 2
TNF-α: Tumor necrosis factor alpha
TRADD: TNFR1-associated death domain protein
TRAF2: TNF receptor associated factor 2

## Footnotes

1 Thermo Fisher Scientific, Waltham, MA, USA

2 Cell Culture Technologies LLC, Gravesano, Ticino, Switzerland

3 GE Healthcare UK Ltd., Amersham Place, Little Chalfont, Buckinghamshire, England

4 Sigma-Aldrich, St. Louis, MO, USA

5 Invitrogen, Carlsbad, CA, USA

6 Molecular Research Center, Inc., Montgomery Road, Cincinnati, OH, USA

7 TaKaRa, Shiga, Japan

8 NIPPON Genetics Co., Ltd., Tokyo, Japan

9 ProteNova, Tokushima, Japan

10 Merck, Summit Drive, Burlington, MA, USA

11 Rockland Immunochemicals, Pottstown, PA, USA

12 Sumitomo Bakelite Co., Ltd., Tokyo, Japan

13 Kingfisher Biotech, Inc., Westgate Drive Suite, Saint Paul, MN, USA

14 Bethyl Laboratories, Inc., P.P. Box, Montgomery, TX, USA

15 GreinerBio-One GmbH, Bad Haller, Kremsmünster, Austria

16 Promega, Fitchburg, WI, USA

17 Roche Diagnostics, Mannheim, Germany
